# Atypical Presentation of Hand, Foot, and Mouth Disease in an Adult

**DOI:** 10.5811/cpcem.2018.3.37686

**Published:** 2018-04-17

**Authors:** Demis N. Lipe, Susan Affleck

**Affiliations:** Martin Army Community Hospital, Department of Emergency Medicine, Fort Benning, Georgia

## CASE PRESENTATION

**Image 1:**
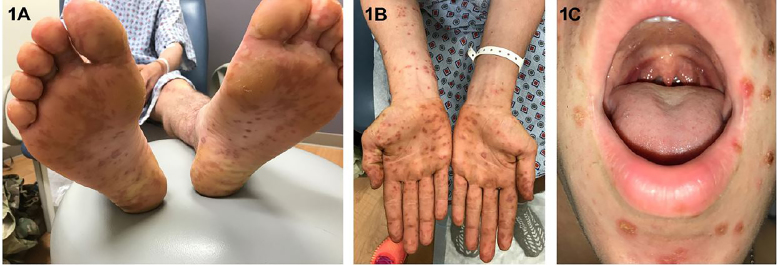
Dermatologic manifestation of atypical hand, foot, and mouth disease, illustrating A) ill-defined, erythematous to violaceous lesions on plantar surface of feet; B) discrete, violaceous lesions extending from palmar surface of hands onto the flexor aspects of the upper extremities; and C) perioral, crusted lesions.

**Image 2:**
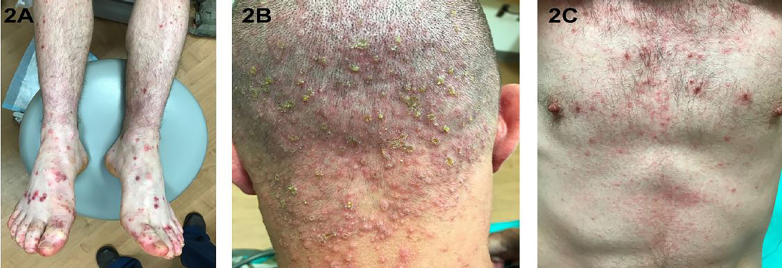
Atypical manifestation of hand, foot, and mouth disease demonstrating A) discrete, purpuric and hemorrhagic lesions of the lower extremities; B) grouped papulo-vesicular lesions with superimposed bacterial colonization; and C) extensive involvement of the torso.

A 21-year-old, active-duty military male presented to the emergency department (ED) with three days of fever of 103^o^F, fatigue, rash and sore throat. The rash was especially painful on his hands and feet. The patient’s history was significant for having spent a week in a field training exercise in a wooded area. Vital signs were normal. Examination revealed purpuric, maculo-papular lesions and erosions of the feet (Image 1A and 2A) and hands extending onto his forearms (Image 1B). He also had crusted erosions periorally and soft palate petechiae (Image 1C). Additionally, there were crusted lesions on the head extending into the neck and torso (Image 2B and 2C).

Initial workup included a rapid antigen streptococcal test, rapid plasma reagin, Rocky Mountain spotted fever (RMSF), coxsackievirus serologies, complete blood count and coagulation studies. The laboratory testing resulted during his ED stay was normal. The RMSF and coxsackievirus serology results returned within the week. The patient was discharged from the ED with a presumptive diagnosis of RMSF on a doxycycline regimen.

## DIAGNOSIS

The patient was later diagnosed with hand, foot, and mouth disease (HFMD) after serology testing was positive for coxsackievirus A6 (CVA6) and the rest of the workup was normal. HFMD typically occurs in children, and historically adults have been asymptomatic.^1^,^2^ With a recent increase in emergence of CVA6, several outbreaks have been reported worldwide.^1^–^4^

Atypical HFMD presents with more variable and severe manifestation such as diffuse rash, purpuric lesions and adult-age predilection.^1^,^4^ Transmission can occur via respiratory secretions, oral-oral, fecal-oral, or contact with fomites.^1^,^2^,^4^ Sharing close living quarters makes military trainees more susceptible to being infected with the virus.^1^ Complications, although rare, can include onychomadesis, bacterial skin superinfection, encephalitis and aseptic meningitis.^2^ The patient continued his doxycycline regimen for the bacterial superinfection and recovered without complications.

**CPC-EM Capsule**

What do we already know about this clinical entity?

Hand, foot, and mouth disease (HFMD) is a common viral illness usually affecting infants and children. Common manifestations are fever and vesicular rash on the hands, feet and buttocks, along with oral ulcers. Common causes of typical HFMD are coxsackievirus A16 and enterovirus 71.

What is the major impact of the image(s)?

These images show the atypical dermatological manifestation of HFMD in an adult, which includes purpuric lesions and more diffuse involvement.

How might this improve emergency medicine practice?

Emergency physicians need to recognize the atypical presentation in adults and include it in the differential diagnosis of purpuric rash involving the extremities. HFMD treatment is conservative, unlike syphilis, Rocky Mountain spotted fever and even Henoch-Schonlein purpura.

Documented patient informed consent and/or Institutional Review Board approval has been obtained and filed for publication of this case report.

## References

[1] Banta J, Lenz B, Pawlak M (2016). Notes from the field: outbreak of hand, foot, and mouth disease caused by coxsackievirus A6 among basic military trainees – Texas, 2015. MMWR Morb Mortal Wkly Rep.

[2] (2017). Hand, Foot, and Mouth Disease (HFMD).

[3] Bian L, Wang Y, Yao X (2015). Coxsackievierus A6: a new emerging pathogen causing hand, foot and mouth disease outbreaks worldwide. Expert Rev Anti Infect Ther.

[4] Ramirez-Fort MK, Downing C, Doan HQ (2014). Coxsackievirus A6 associated hand, foot and mouth disease in adults: clinical presentation and review of the literature. J Clin Virol.

